# Supplementary material for: Identification of genetic loci in lettuce mediating quantitative resistance to fungal pathogens
Source: Theor Appl Genet. 2022 Jun 8;135(7):2481–500. doi: 10.1007/s00122-022-04129-5 (PMC9271113; doi:10.1007/s00122-022-04129-5)
Supplement: Supplementary file 10 — Supplementary file10 (PPTX 1505 KB) [file 122_2022_4129_MOESM10_ESM.pptx]

## Slide 1
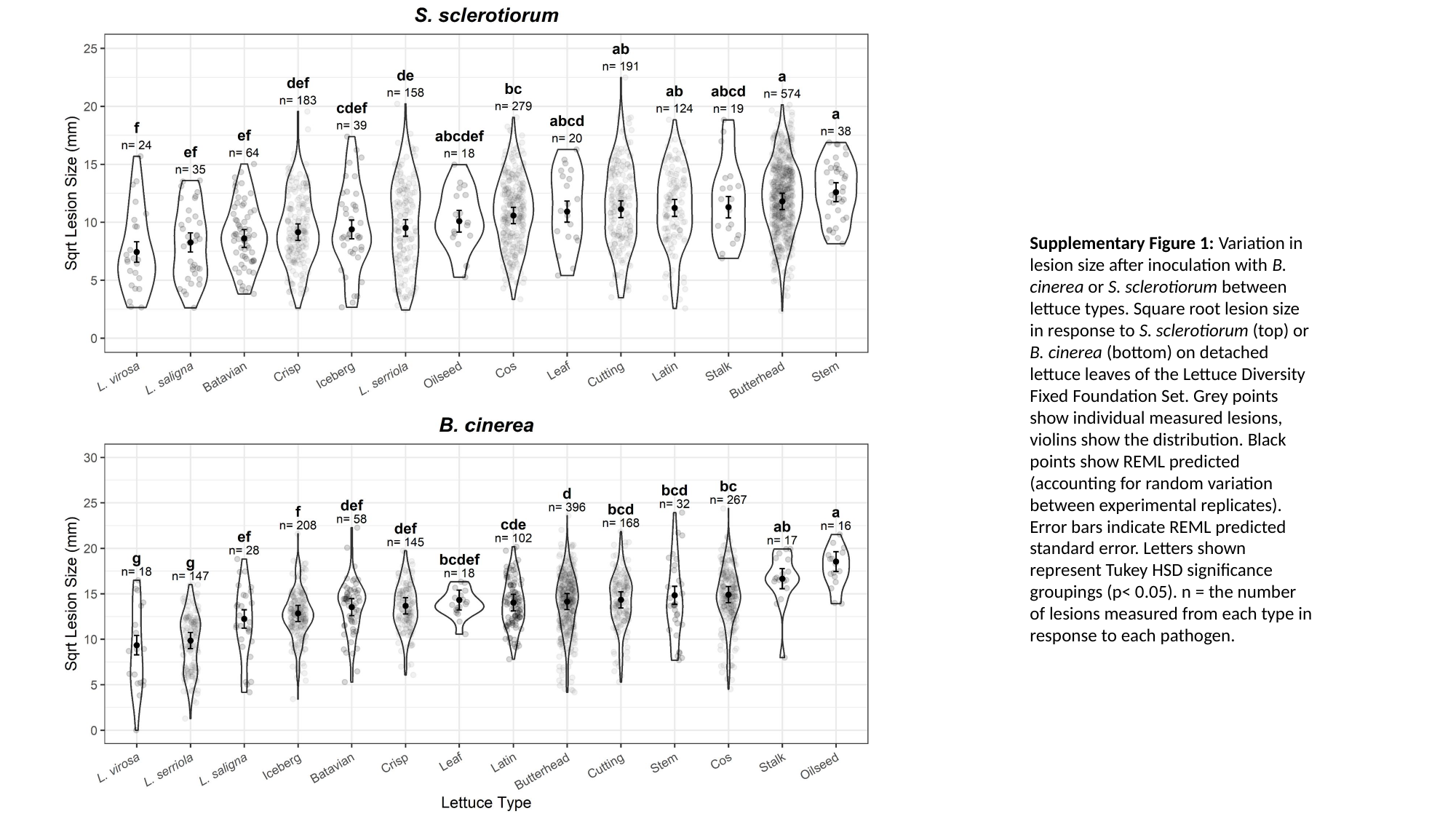

Supplementary Figure 1: Variation in lesion size after inoculation with B. cinerea or S. sclerotiorum between lettuce types. Square root lesion size in response to S. sclerotiorum (top) or B. cinerea (bottom) on detached lettuce leaves of the Lettuce Diversity Fixed Foundation Set. Grey points show individual measured lesions, violins show the distribution. Black points show REML predicted (accounting for random variation between experimental replicates). Error bars indicate REML predicted standard error. Letters shown represent Tukey HSD significance groupings (p< 0.05). n = the number of lesions measured from each type in response to each pathogen.
