# Supplementary material for: Identification of genetic loci in lettuce mediating quantitative resistance to fungal pathogens
Source: Theor Appl Genet. 2022 Jun 8;135(7):2481–500. doi: 10.1007/s00122-022-04129-5 (PMC9271113; doi:10.1007/s00122-022-04129-5)
Supplement: Supplementary file 11 — Supplementary file11 (PPTX 33267 KB) [file 122_2022_4129_MOESM11_ESM.pptx]

## Slide 1
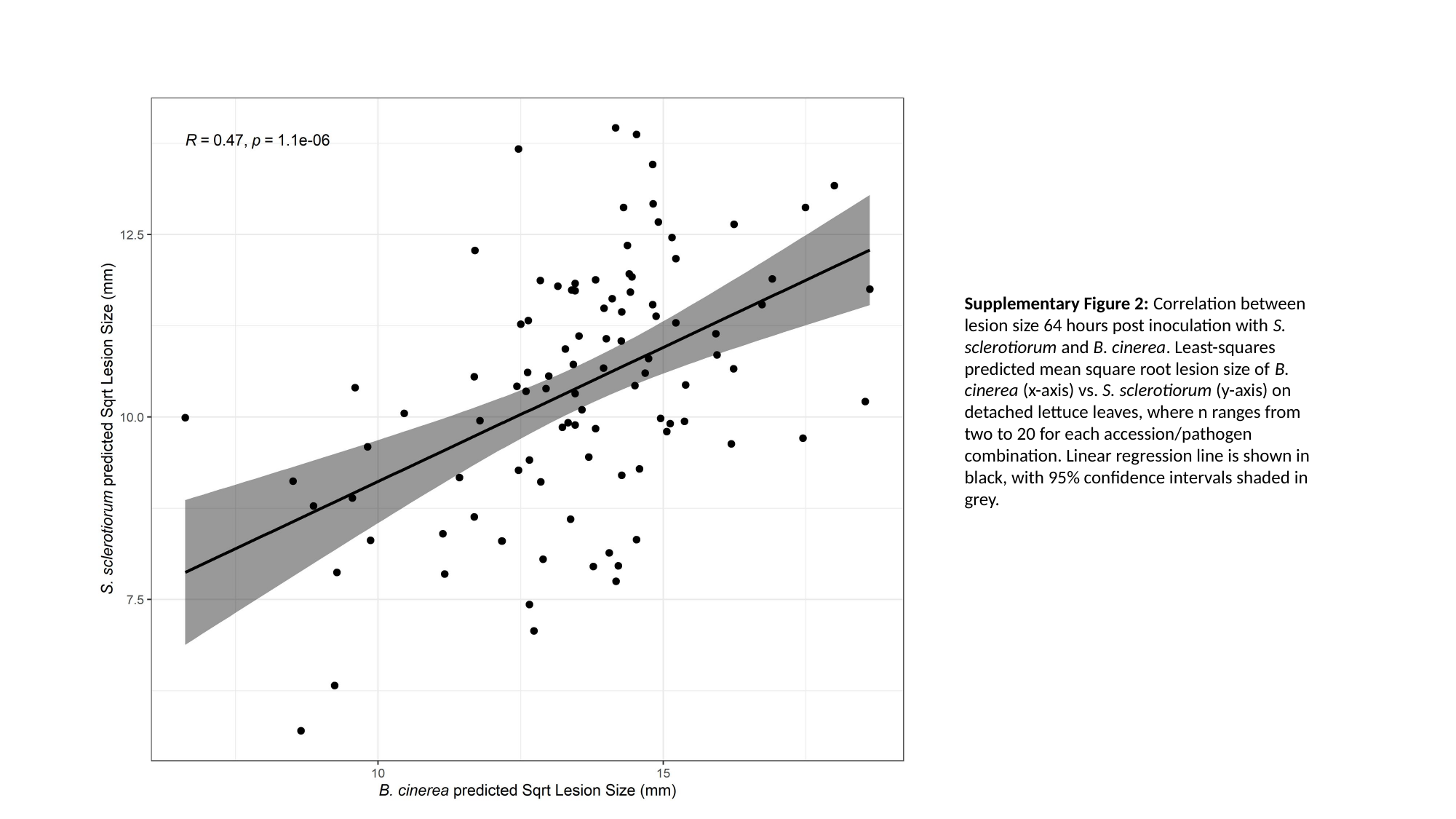

Supplementary Figure 2: Correlation between lesion size 64 hours post inoculation with S. sclerotiorum and B. cinerea. Least-squares predicted mean square root lesion size of B. cinerea (x-axis) vs. S. sclerotiorum (y-axis) on detached lettuce leaves, where n ranges from two to 20 for each accession/pathogen combination. Linear regression line is shown in black, with 95% confidence intervals shaded in grey.
