# Supplementary material for: Identification of genetic loci in lettuce mediating quantitative resistance to fungal pathogens
Source: Theor Appl Genet. 2022 Jun 8;135(7):2481–500. doi: 10.1007/s00122-022-04129-5 (PMC9271113; doi:10.1007/s00122-022-04129-5)
Supplement: Supplementary file 12 — Supplementary file12 (PPTX 1831 KB) [file 122_2022_4129_MOESM12_ESM.pptx]

## Slide 1
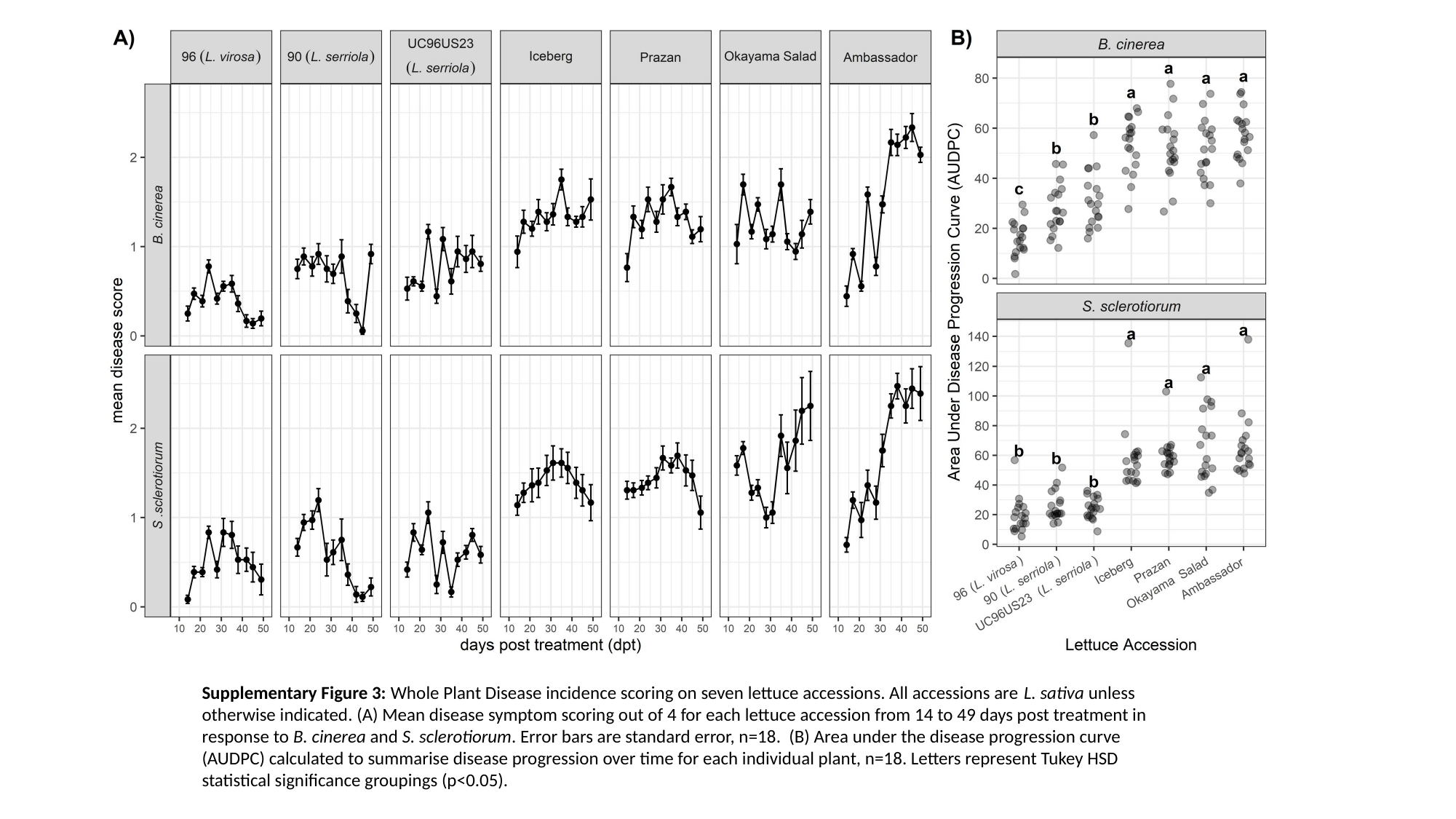

Supplementary Figure 3: Whole Plant Disease incidence scoring on seven lettuce accessions. All accessions are L. sativa unless otherwise indicated. (A) Mean disease symptom scoring out of 4 for each lettuce accession from 14 to 49 days post treatment in response to B. cinerea and S. sclerotiorum. Error bars are standard error, n=18. (B) Area under the disease progression curve (AUDPC) calculated to summarise disease progression over time for each individual plant, n=18. Letters represent Tukey HSD statistical significance groupings (p<0.05).
