# Supplementary material for: Identification of genetic loci in lettuce mediating quantitative resistance to fungal pathogens
Source: Theor Appl Genet. 2022 Jun 8;135(7):2481–500. doi: 10.1007/s00122-022-04129-5 (PMC9271113; doi:10.1007/s00122-022-04129-5)
Supplement: Supplementary file 13 — Supplementary file13 (PPTX 28515 KB) [file 122_2022_4129_MOESM13_ESM.pptx]

## Slide 1
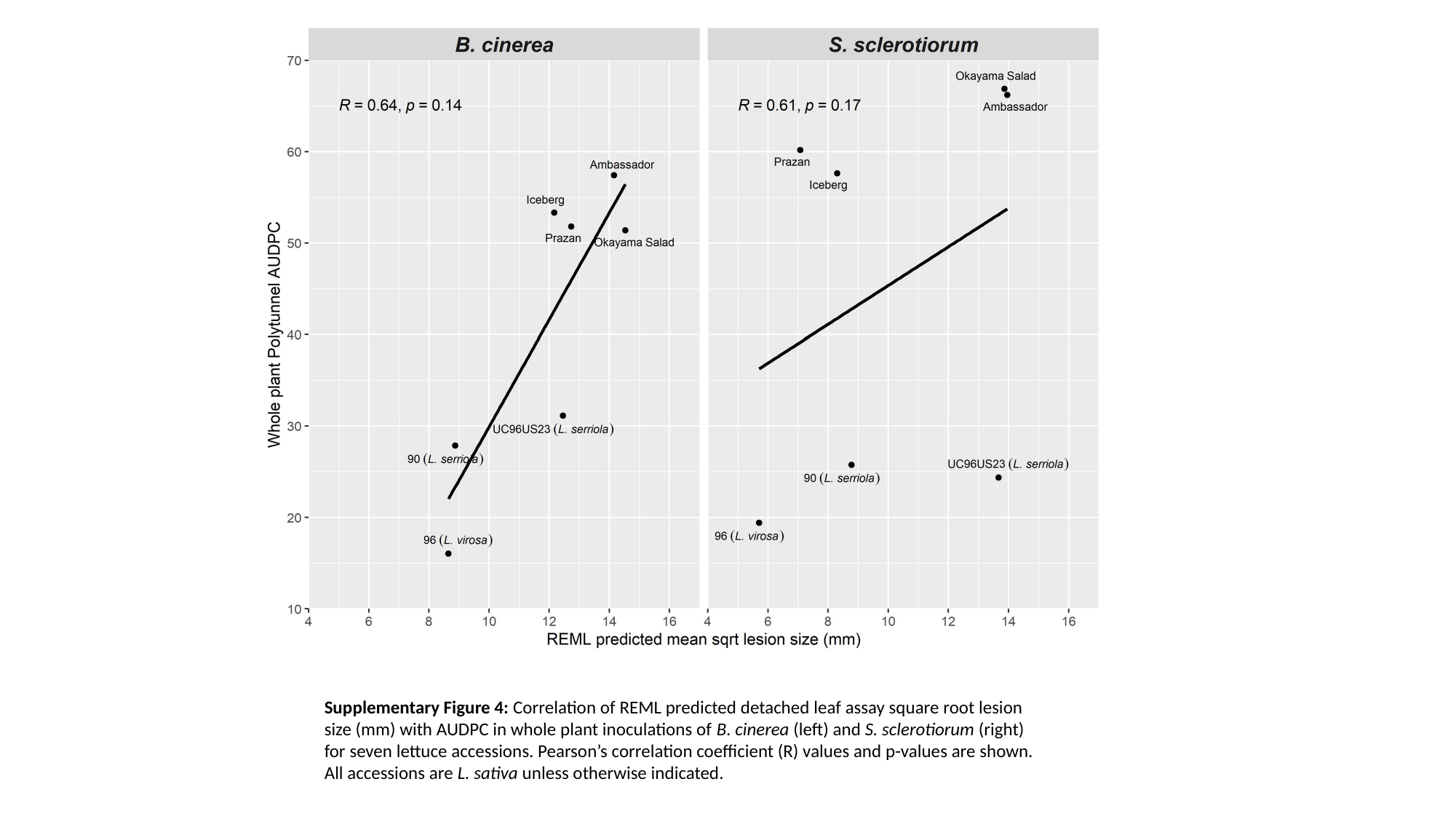

Supplementary Figure 4: Correlation of REML predicted detached leaf assay square root lesion size (mm) with AUDPC in whole plant inoculations of B. cinerea (left) and S. sclerotiorum (right) for seven lettuce accessions. Pearson’s correlation coefficient (R) values and p-values are shown. All accessions are L. sativa unless otherwise indicated.
