# Supplementary material for: Identification of genetic loci in lettuce mediating quantitative resistance to fungal pathogens
Source: Theor Appl Genet. 2022 Jun 8;135(7):2481–500. doi: 10.1007/s00122-022-04129-5 (PMC9271113; doi:10.1007/s00122-022-04129-5)
Supplement: Supplementary file 14 — Supplementary file14 (PPTX 1241 KB) [file 122_2022_4129_MOESM14_ESM.pptx]

## Slide 1
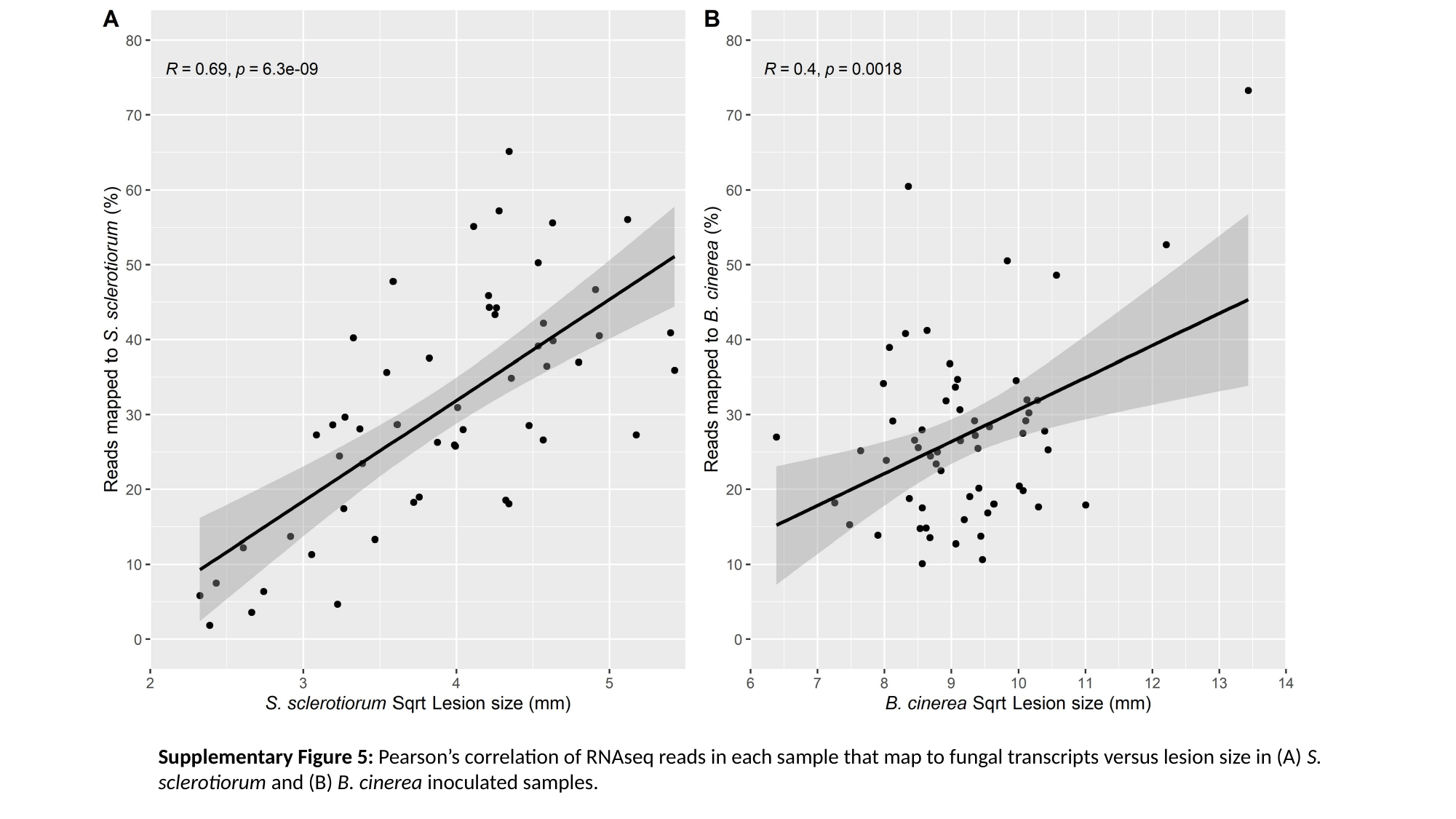

#
Supplementary Figure 5: Pearson’s correlation of RNAseq reads in each sample that map to fungal transcripts versus lesion size in (A) S. sclerotiorum and (B) B. cinerea inoculated samples.
