# Supplementary material for: Identification of genetic loci in lettuce mediating quantitative resistance to fungal pathogens
Source: Theor Appl Genet. 2022 Jun 8;135(7):2481–500. doi: 10.1007/s00122-022-04129-5 (PMC9271113; doi:10.1007/s00122-022-04129-5)
Supplement: Supplementary file 15 — Supplementary file15 (PPTX 1899 KB) [file 122_2022_4129_MOESM15_ESM.pptx]

## Slide 1
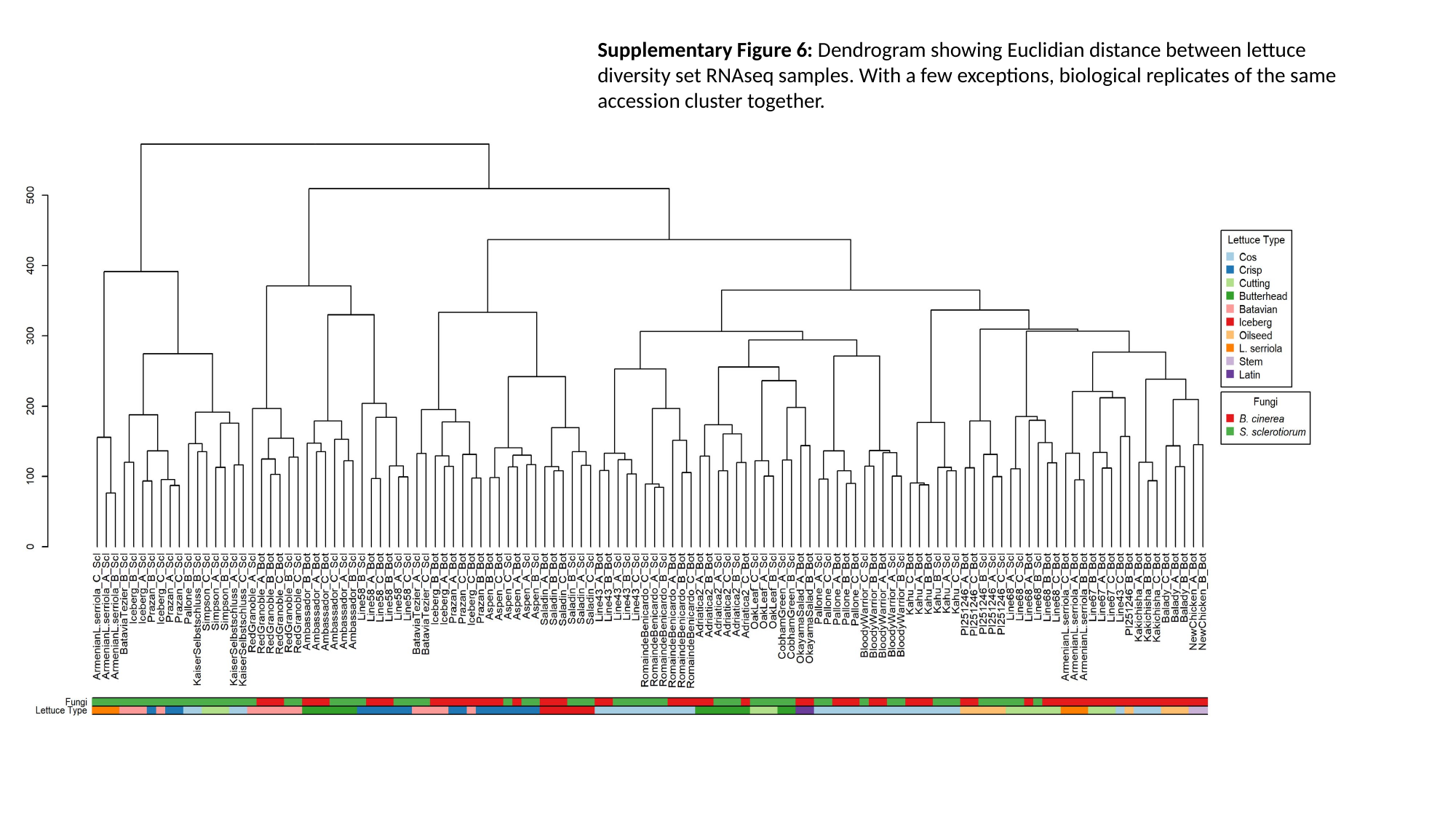

Supplementary Figure 6: Dendrogram showing Euclidian distance between lettuce diversity set RNAseq samples. With a few exceptions, biological replicates of the same accession cluster together.
#
