# Supplementary material for: Identification of genetic loci in lettuce mediating quantitative resistance to fungal pathogens
Source: Theor Appl Genet. 2022 Jun 8;135(7):2481–500. doi: 10.1007/s00122-022-04129-5 (PMC9271113; doi:10.1007/s00122-022-04129-5)
Supplement: Supplementary file 16 — Supplementary file16 (PPTX 2978 KB) [file 122_2022_4129_MOESM16_ESM.pptx]

## Slide 1
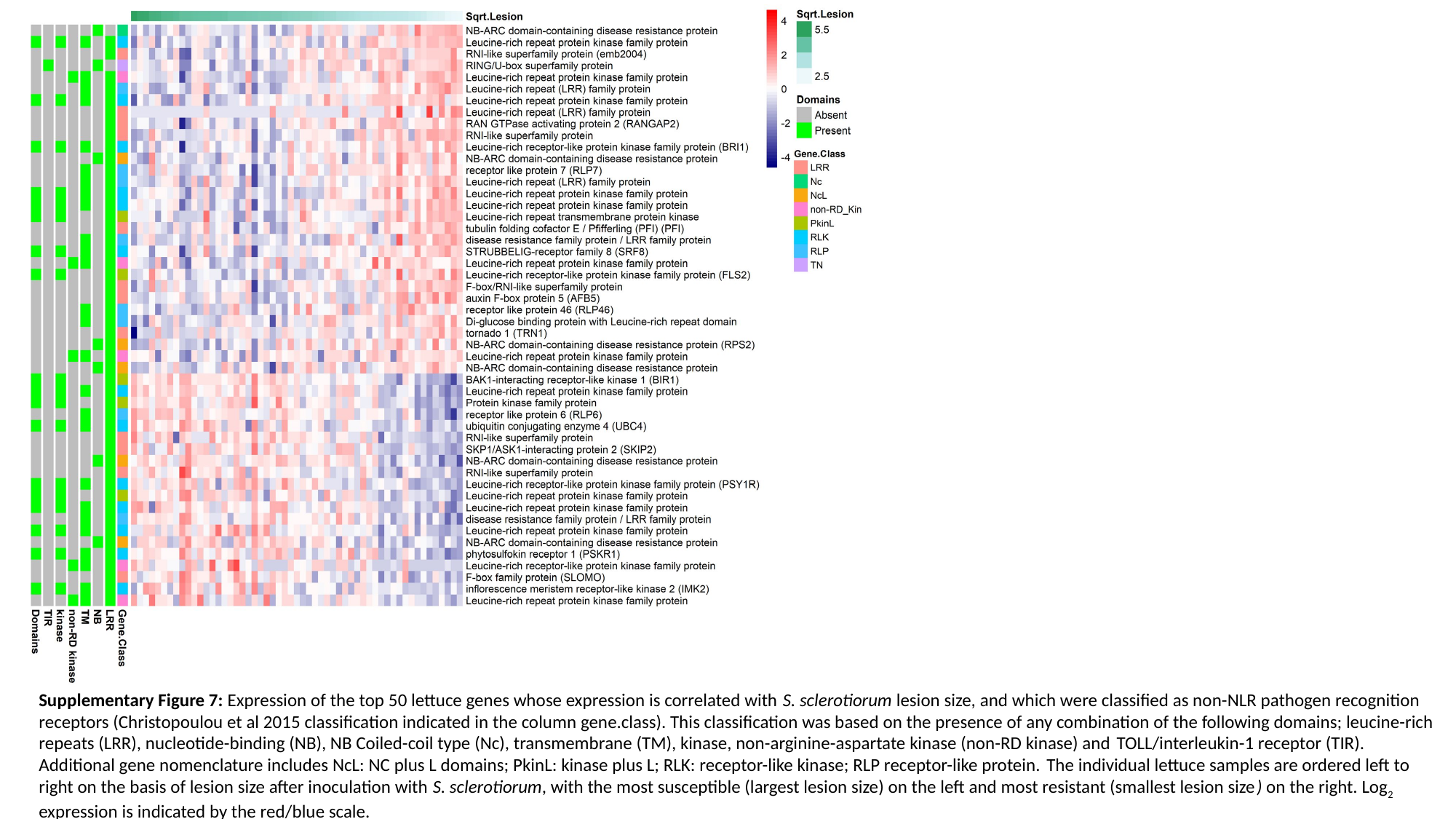

Supplementary Figure 7: Expression of the top 50 lettuce genes whose expression is correlated with S. sclerotiorum lesion size, and which were classified as non-NLR pathogen recognition receptors (Christopoulou et al 2015 classification indicated in the column gene.class). This classification was based on the presence of any combination of the following domains; leucine-rich repeats (LRR), nucleotide-binding (NB), NB Coiled-coil type (Nc), transmembrane (TM), kinase, non-arginine-aspartate kinase (non-RD kinase) and TOLL/interleukin-1 receptor (TIR). Additional gene nomenclature includes NcL: NC plus L domains; PkinL: kinase plus L; RLK: receptor-like kinase; RLP receptor-like protein. The individual lettuce samples are ordered left to right on the basis of lesion size after inoculation with S. sclerotiorum, with the most susceptible (largest lesion size) on the left and most resistant (smallest lesion size) on the right. Log2 expression is indicated by the red/blue scale.
