# Supplementary material for: Identification of genetic loci in lettuce mediating quantitative resistance to fungal pathogens
Source: Theor Appl Genet. 2022 Jun 8;135(7):2481–500. doi: 10.1007/s00122-022-04129-5 (PMC9271113; doi:10.1007/s00122-022-04129-5)
Supplement: Supplementary file 17 — Supplementary file17 (PPTX 738 KB) [file 122_2022_4129_MOESM17_ESM.pptx]

## Slide 1
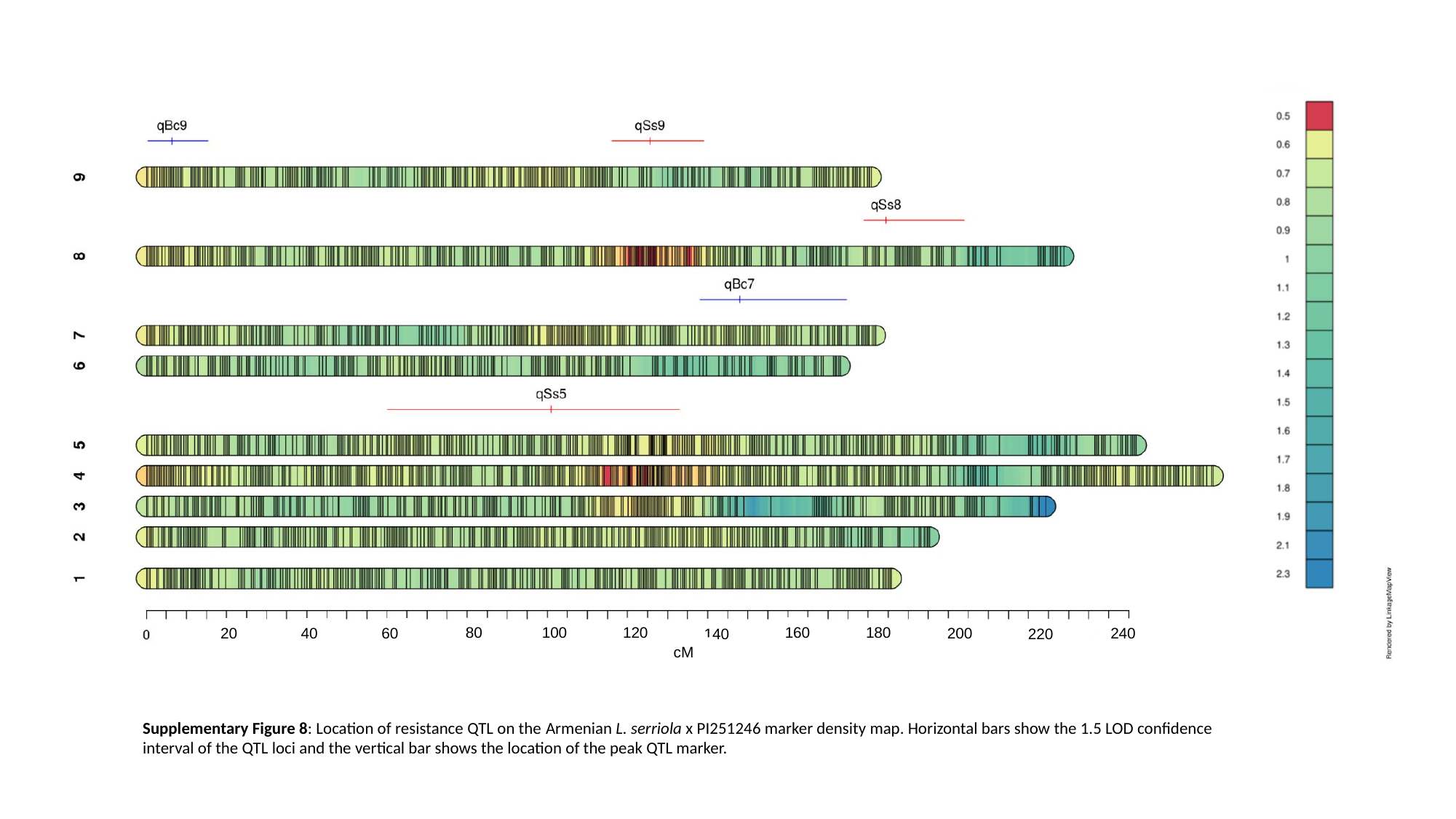

#
160
180
120
80
100
20
40
60
200
240
140
220
cM
Supplementary Figure 8: Location of resistance QTL on the Armenian L. serriola x PI251246 marker density map. Horizontal bars show the 1.5 LOD confidence interval of the QTL loci and the vertical bar shows the location of the peak QTL marker.
