# Supplementary material for: Identification of genetic loci in lettuce mediating quantitative resistance to fungal pathogens
Source: Theor Appl Genet. 2022 Jun 8;135(7):2481–500. doi: 10.1007/s00122-022-04129-5 (PMC9271113; doi:10.1007/s00122-022-04129-5)
Supplement: Supplementary file 18 — Supplementary file18 (PPTX 1421 KB) [file 122_2022_4129_MOESM18_ESM.pptx]

## Slide 1
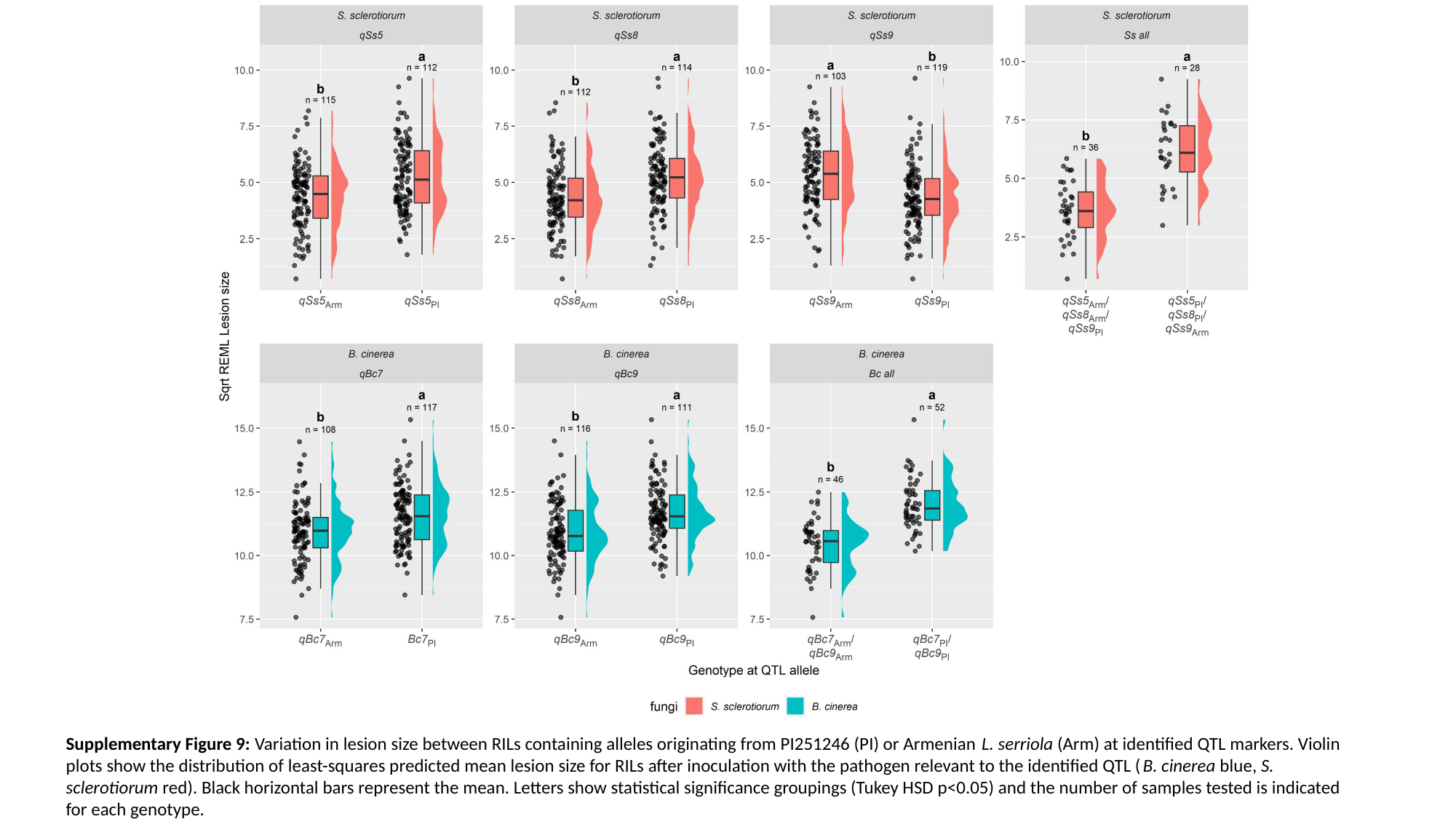

Supplementary Figure 9: Variation in lesion size between RILs containing alleles originating from PI251246 (PI) or Armenian L. serriola (Arm) at identified QTL markers. Violin plots show the distribution of least-squares predicted mean lesion size for RILs after inoculation with the pathogen relevant to the identified QTL (B. cinerea blue, S. sclerotiorum red). Black horizontal bars represent the mean. Letters show statistical significance groupings (Tukey HSD p<0.05) and the number of samples tested is indicated for each genotype.
