# Supplementary material for: Identification of genetic loci in lettuce mediating quantitative resistance to fungal pathogens
Source: Theor Appl Genet. 2022 Jun 8;135(7):2481–500. doi: 10.1007/s00122-022-04129-5 (PMC9271113; doi:10.1007/s00122-022-04129-5)
Supplement: Supplementary file 19 — Supplementary file19 (PPTX 1732 KB) [file 122_2022_4129_MOESM19_ESM.pptx]

## Slide 1
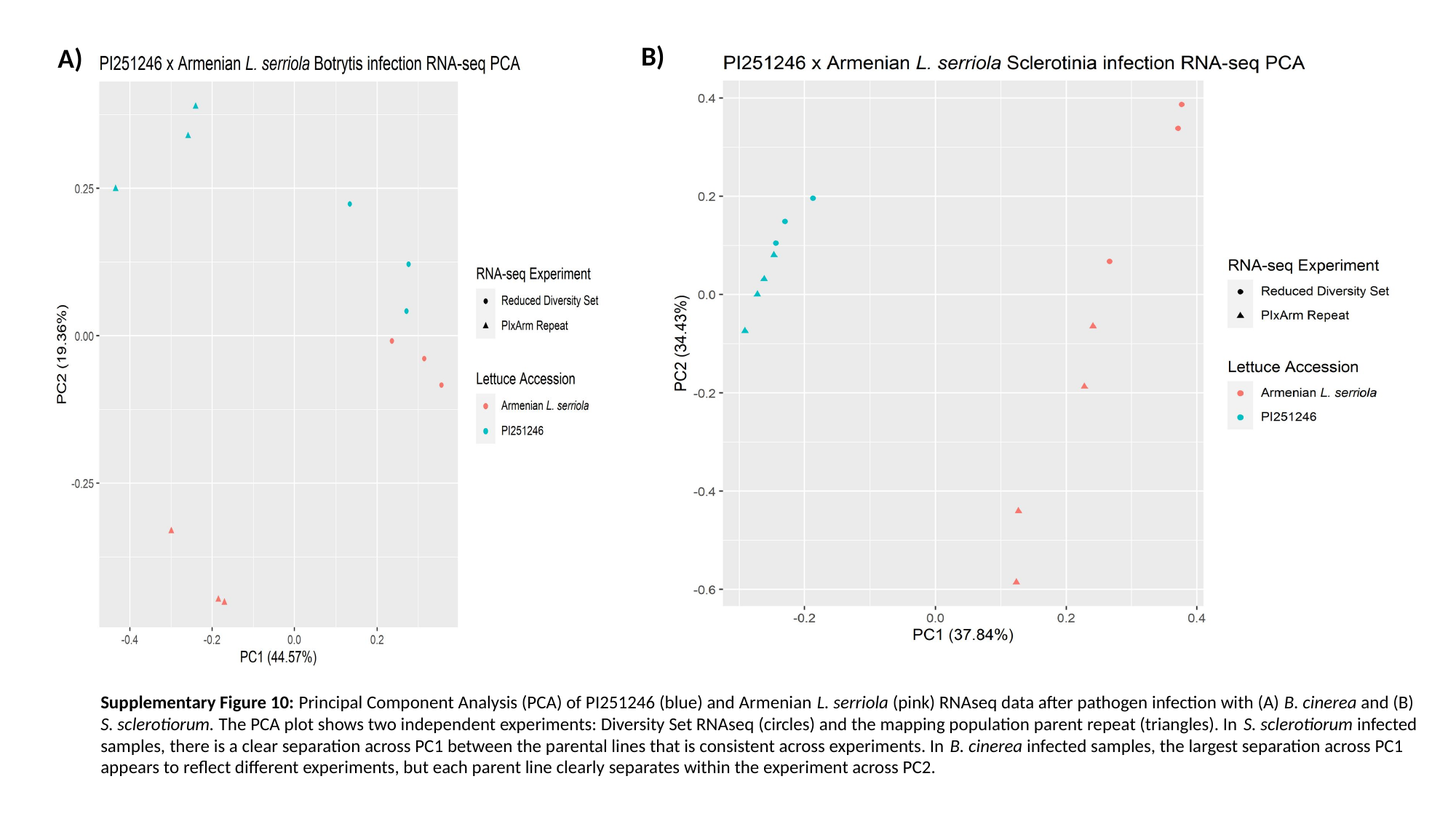

B)
A)
Supplementary Figure 10: Principal Component Analysis (PCA) of PI251246 (blue) and Armenian L. serriola (pink) RNAseq data after pathogen infection with (A) B. cinerea and (B) S. sclerotiorum. The PCA plot shows two independent experiments: Diversity Set RNAseq (circles) and the mapping population parent repeat (triangles). In S. sclerotiorum infected samples, there is a clear separation across PC1 between the parental lines that is consistent across experiments. In B. cinerea infected samples, the largest separation across PC1 appears to reflect different experiments, but each parent line clearly separates within the experiment across PC2.
